# Sulphonal in Diabetes

**Published:** 1890-07-05

**Authors:** 


					SULPHONAL IN DIABETES.
OU1.FH0NAL IN unsatisfactory
It is not long since that we pom e &Q occasional
? ar&cter of the much belauded su p o ^ the patient,
ypnotic on account of the tardiness o ^ ought to he
*>t, not falling into a sound throughout
flaking of rising, and f<eeling"its habitual
enext day; as well as the real dane physiological
or continued use arising from the fac trea depressing
JBoa i8 exerted prima?ily 0n the Bpmal^tres,^ J ^ ^
6 motor and disturbing the co-ordina 1 g .g a 8eri0us
Cord before affecting those of thesensorium. ^ employ-
c?HBideration, and should induce one to e administra-
? ^ ^ ln c^r?nic diseases requiring its con inn ascribe
r*?n- Yet to its action on the spinal centres o him in
<1 e e2ects observed by Dr. Casarelli, and reP _,, volume
_.e ^iuw(a Generate di Glinica, in reducing v.etes, effects
,e urine and the excretion of sugar 1D . i:et were re-
*b}ch he found equally marked, whether the c in
ricted or not, different from the ordinary. ? three
t8ea of from one to two, or if the patient bore it we^
^ammes in the 24 hours, for several days in succe

				

